# Teaching medical undergraduates Skills for Eye Examination: Is Flipped Classroom with Mental Rehearsal as Effective as Face-to-Face Teaching?

**DOI:** 10.5334/pme.2234

**Published:** 2026-08-24

**Authors:** Chee Chew Yip, Zheng Xian Thng, Yew Sen Yuen, Ray Manotosh, Jianbin Ding, Woon Teck Clement Tan, Johnson Choon Hwai Tan, Dujeepa Samareasekera

**Affiliations:** 1 Ophthalmology & Visual Sciences Department, Khoo Teck Puat Hospital, Singapore; 2 Yong Loo Lin School of Medicine, National University of Singapore, Singapore; 3 Education Development Office, Khoo Teck Puat Hospital, Singapore; 4 Ophthalmology Department, National University Hospital, Singapore; 5 Ophthalmology Department, Tan Tock Seng Hospital, Singapore; 6 Raffles Hospital, Singapore

## Abstract

**Introduction:**

The quality of face-to-face teaching (F2FT) of eye-examination is often suboptimal amidst busy clinics as it demands faculty time for teaching and feedback. Flipped Classroom (FC) and Mental Rehearsal (MR) are established methods to teach clinical skills. A learning module (Supplementary Clinical Ophthalmology Preparatory E-learning [SCOPE]) that combines FC and MR may address this practical problem. This quasi-experimental study compared the efficacy and Faculty Contact Time of SCOPE against F2FT in teaching fourth-year medical undergraduates eye-examination.

**Method:**

The SCOPE group (n = 95) received the same content as F2FT but delivered in deliberate sequence: e learning (instructional videos and Video Modelling Examples), followed by structured, audio guided MR, then face-to-face practice with feedback. The F2FT group (n = 51) learned in a single face-to-face session with live faculty demonstration and Video Modelling Examples, without instructional videos or MR.

**Results:**

Baseline pre-intervention mean Multiple Choice Questions (MCQ) scores were comparable (22.31 versus 22.24, independent t-test, p = .94). Post-learning cognitive assessments found the SCOPE group to have greater gain in total MCQ scores with higher estimated marginal means (p < .001), and higher mean End-of-Rotation Test scores (43.37 versus 40.85, independent t-test, p = .048). On psychomotor assessments, the SCOPE group had superior Micro-CEX performances (three skills, all *p* < .001, Fisher’s Exact test). SCOPE also shortened Faculty Contact Time, saving on average 57.48 minutes (*p* < .001, independent t-test).

**Discussion:**

SCOPE improved psychomotor and cognitive performances, and was more faculty time-efficient than F2FT in teaching eye examination skills. These positive results may be attributed to the synergistic effects of its multiple learning components. SCOPE, which combined FC and MR, may better facilitate clinical skill development through deliberate practice than F2FT.

## Introduction

Ophthalmology is part of the core curriculum for medical undergraduates, with students expected to master Skills for Eye Examination (SEE). These skills require both procedural and cognitive competence: learners must perform examination steps accurately while interpreting clinical signs and making diagnostic decisions. However, traditional Face-to-Face Teaching (F2FT) faces challenges. Limited Faculty Contact Time in high-volume clinics and Singapore’s brief two-week ophthalmology rotation constrain SEE learning. Combined with limited resources for students who need additional time or support, this underscores the need for more effective teaching methods. Flipped Classroom (FC) and Mental Rehearsal (MR) are established learning approaches and combining them into a novel FC-MR model may address these issues. However, there is limited research on its effectiveness for clinical skills teaching.

### Theoretical Framework for a Combined Model

FC, involving self-learning followed by faculty led F2FT sessions, has been shown to be as effective or superior to traditional methods [1]. MR, an independent mental simulation technique, enhances performance in surgical education [2,3]. Combining FC and MR addresses F2FT challenges in teaching SEE by shifting some learning to the student. The integrated FC-MR model aligns with the Deliberate Practice Theory, emphasizing structured, purposeful training to improve performance [4].

The model incorporates the three key elements of Deliberate Practice: well-defined tasks, repetitive practice, and timely feedback. It operationalizes Deliberate Practice Theory by structuring student learning around clear examination tasks (e.g., pupil examination or visual field examination), repeated through guided mental rehearsal and reinforced with faculty feedback during practice [4,5,6]. This purposeful cycle of rehearsal, application, and correction mirrors the key tenets of deliberate practice.

While MR and e-learning have shown benefits [7,8], further research is needed to evaluate the effectiveness of the FC-MR model in teaching clinical skills.

### Flipped Classroom

FC addresses the resource and feedback limitations of traditional F2FT by combining self-paced e-learning (narrated lectures, videos, multimedia) with F2FT practice and feedback. It reduces the need for extensive Faculty Contact Time and its benefits are enhanced by adding quizzes [9,10,11].

FC improves student motivation, understanding, communication, critical thinking, and test performance in ophthalmology [12], while fostering problem-solving and teamwork [13]. FC also enhances psychomotor skills without increasing faculty time [14]. Instructional videos improve examination techniques and clinical performance [15], and e-learning primes students for better physical examination in F2FT session [16]. FC promotes self-regulated, active learning by encouraging learners to take ownership of their progress. It enables repeated review of complex content at one’s own pace, enhancing retention and understanding [17].

### Mental Rehearsal

MR uses a mental imagery script to simulate sensory experiences during a task [18]. Based on the Motor Simulation Theory, MR activates brain motor systems similar to actual actions [19,20], with changes in the brain resembling those after physical practice [21]. MR enhances learning of complex tasks like cystoscopy and laparoscopic cholecystectomy [3,22]. Meta-analysis shows MR effectively supplements physical training, especially in experienced learners [23], and improves cognitive tasks like decision-making and problem-solving [2], making it suitable for teaching SEE. MR allows independent practice at one’s own pace. Two meta-analyses confirm its positive impact on skill performance [24,25], with efficacy increased by prior experience [26]. Modelling examples can also improve MR’s effectiveness [27,28]. MR supports emotional regulation by reducing anxiety and enhancing confidence before critical tasks [29]. It reinforces procedural memory vital for fine motor skills and activates mirror neurons, promoting observational learning and empathy in patient-centered care [30,31]. Easily adaptable to virtual environments, MR enhances accessibility and engagement, making it a strong, evidence-based intervention for developing both technical and non-technical skills.

### Current Study

FC and MR are instructional approaches that are not inherently forms of deliberate practice. However, when intentionally designed and sequenced, they can support key elements of Deliberate Practice Theory, including well-defined tasks, repeated practice, and focused feedback.

The knowledge gap is not whether FC or MR can work in isolation. Rather, it is whether a combined FC-MR model can improve learning of clinical examination skills more than conventional F2FT in terms of psychomotor execution and cognitive interpretation, while reducing dependence on faculty time. Existing ophthalmology FC studies have mainly examined clerkship teaching [12,13], rather than an integrated model for teaching examination skills themselves. Likewise, although MR and e-learning have shown benefits in procedural education [24,25], there has been little direct evaluation of a deliberately sequenced FC-MR model for teaching clinical examination skills with procedural and cognitive elements. This study addresses that gap.

We therefore developed the Supplementary Clinical Ophthalmology Preparatory E-learning (SCOPE) module, which combines FC and MR to teach SEE. SCOPE operationalises Deliberate Practice Theory by structuring learning into self-paced online study, guided MR, and focused face-to-face practice with feedback. The aim of this study was to compare SCOPE, a FC-MR model, with conventional F2FT for teaching SEE to medical undergraduates.

We have two main hypotheses:

1. SCOPE is more efficacious than F2FT in teaching medical undergraduates SEE to improve cognitive and psychomotor skill performances.
2. SCOPE reduces Faculty Contact Time compared to F2FT.

This study aimed to compare the efficacy and faculty efficiency of the SCOPE module with traditional F2FT in teaching undergraduate SEE. The primary outcomes were improvement in cognitive knowledge (Multiple Choice Question and End-of-Rotation Test scores) and psychomotor skills (Micro-CEX performance). The secondary outcome was Faculty Contact Time required to achieve the primary outcomes.

## Methods

### Setting and Design

This IRB-approved study (National University of Singapore, S-18-301) was conducted at three accredited undergraduate training centres over seven months. Due to manpower and facility constraints, it was not possible for each centre to deliver two forms of teaching and centres were not randomised. Centre 1 (Khoo Teck Puat Hospital, n = 51) and Centre 2 (National University Hospital, n = 44) delivered SCOPE, while Centre 3 (Tan Tock Seng Hospital, n = 51) delivered F2FT. Students followed pre-assigned institutional rotations determined by the university administration; investigators had no role in assigning students to groups.

The study used a quasi-experimental, non-equivalent pre–post design, enrolling a pragmatic census of all eligible fourth-year students (SCOPE n = 95; F2FT n = 51), with analyses adjusted for centre where applicable. Three SEE were taught—pupil, visual field, and extra-ocular movement examinations—selected for their alignment with both cognitive reasoning and psychomotor execution.

### Participants

One hundred and forty-six fourth-year medical students from the National University of Singapore, undergoing a 2-week Ophthalmology rotation, were recruited with informed consent. All students had completed prior clinical rotations in Internal Medicine, Family Medicine, and General Surgery. Before the study, they attended a half-day didactic session on eye anatomy, theoretical aspects of various SEE (e.g., indications and basis for performing SEE), and common systemic diseases with ocular manifestations. The lectures did not teach the techniques of SEE (psychomotor domain).

### Materials

**Figure 1:**
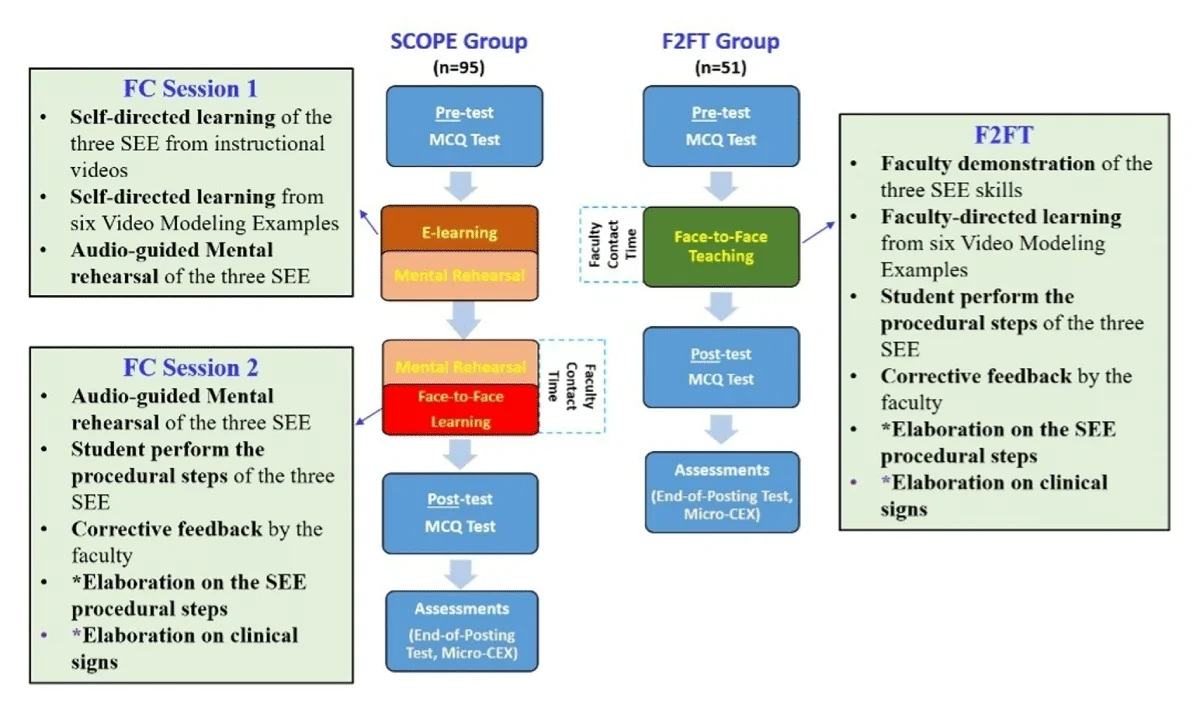
The educational contents and teaching methods are standardised using a teaching script.

Figure 1 shows the interventions, instruments, and procedures in both groups. The SCOPE group had two consecutive FC sessions (e-learning and face-to-face learning), while the F2FT group had one F2FT session. Table 1 provides a summarized description of each learning material and assessment instrument used for the study.

**Table 1:** Table summarizing the teaching materials and assessment instruments used during the study.


ITEM	DESCRIPTION

**Instructional Video**	10-minute video with step-by-step explanation of the eye examination skill of interest, focusing on key checkpoints and pitfalls. Three in total, covering all three skills of interest.

**Mental Rehearsal (MR) Audio**	10-minute expert narrative of all procedural steps involved in the eye examination skill of interest, emphasizing landmarks, decision points, and errors to avoid. Three in total, covering all three skills of interest.

**Video Modelling Examples (VME)**	20-minute video illustrating the problem-solving and diagnostic application of the eye examination skill of interest. Three in total, covering all three skills of interest.

**Multiple Choice Question (MCQ) Test**	50 item test delivered over 1.5hr. Questions assessed knowledge application, diseases diagnosis, and clinical reasoning. Two sets of questions in total, delivered pre- and post-teaching.

**End-of-Rotation Test**	6 short answer questions delivered over 1hr. Questions were vignette-based, assessing clinical reasoning and problem solving using authentic case examples.

**Micro-Clinical Evaluation eXercise**	Single workplace assessment over 10 minutes. Participants examined real patients and were assessed based on accuracy, speed, and time-sharing abilities.


SCOPE’s learning objectives were mapped to the same standardized teaching script used in F2FT. From this script, ophthalmology faculty produced and peer-reviewed instructional videos (expert step-by-step demonstrations), Video Modelling Examples (VMEs), and audio-guided MR scripts to ensure fidelity to the procedural steps and clinical signs taught in routine instruction. Accordingly, SCOPE closely mirrors faculty teaching in F2FT, with the key differences being the structured, audio-guided MR and the deliberate sequencing of activities (e-learning → MR → F2F).

### A. Interventions for the SCOPE Group

#### Flipped Classroom Session 1 (Day 1, 3 hours, standardized self-directed e-learning)

Students learned three SEE sequentially—SEE-1 (pupil), SEE-2 (visual field), and SEE-3 (extra-ocular movements). For each SEE they completed: (1) a 10-min instructional video (expert step-by-step with key checkpoints and pitfalls); (2) a 10-min audio-guided MR—scripted mental imagery of all procedural steps emphasizing landmarks, decision points, and errors to avoid; and (3) 20-min VME illustrating problem-solving and diagnostic application (e.g., pupil findings prompting imaging and diagnosis of an intracranial aneurysm). Materials were accessible only within the 3-hour window, during which students could pause/replay, take breaks, and consult resources/peers, but had no access outside. To prevent novices from reinforcing errors, MR immediately followed the expert video for step-by-step guidance, and was followed the next day by supervised practice with corrective feedback.

#### Flipped Classroom Session 2 (Day 2, Face-to-Face)

A brief audio-guided MR refresh preceded supervised practice of each SEE, with immediate, performance-specific feedback and structured student elaboration using a standardized teaching script (procedural steps, clinical signs, guiding questions). Faculty with more than 5 years of teaching experience were trained on the script, study instruments, and feedback delivery. Activities were repeated for all three SEE with 10-minute breaks.

The two-session, consecutive-day schedule (introducing spacing/recall) was intentional and standardized across SCOPE cohorts. Time-on-task was controlled by fixing Session-1 at 3 hours and measuring Faculty Contact Time separately for Session-2; out-of-session access to SCOPE materials was blocked.

### B. Interventions for the F2FT Group

A single F2F session using the same standardized teaching script: live faculty demonstration of procedural steps, supervised practice with just-in-time feedback, and video-based teaching with VMEs (paused/replayed as needed). Importantly, F2FT students did not view the three instructional videos; faculty covered that content via live demonstration. No audio-guided MR was used. This preserves the pivotal difference between groups (SCOPE = instructional videos + MR + VMEs + F2F; F2FT = live demonstration + VMEs + F2F).

### C. Instruments

**Multiple Choice Question (MCQ) Test** (1.5 hours): This 50-item test, using authentic case scenarios, assessed knowledge application, disease diagnosis, and clinical reasoning [32]. Pre- and post-tests measured baseline and gained knowledge. The test was divided into three sections, with half focusing on procedural knowledge and examination steps (e.g., adapting techniques), and the remainder on clinical reasoning (e.g., interpreting signs like cranial nerve III palsy). Answers were explained post-test to facilitate learning.

**End-of-Rotation Test** (1 hour): Six vignette-based short-answer questions assessed clinical reasoning and problem-solving [32]. Each vignette depicted a pathological eye condition; students provided diagnosis, differentials, key signs, investigations, and management, assessing the cognitive domain of SEE.

**Figure 2:**
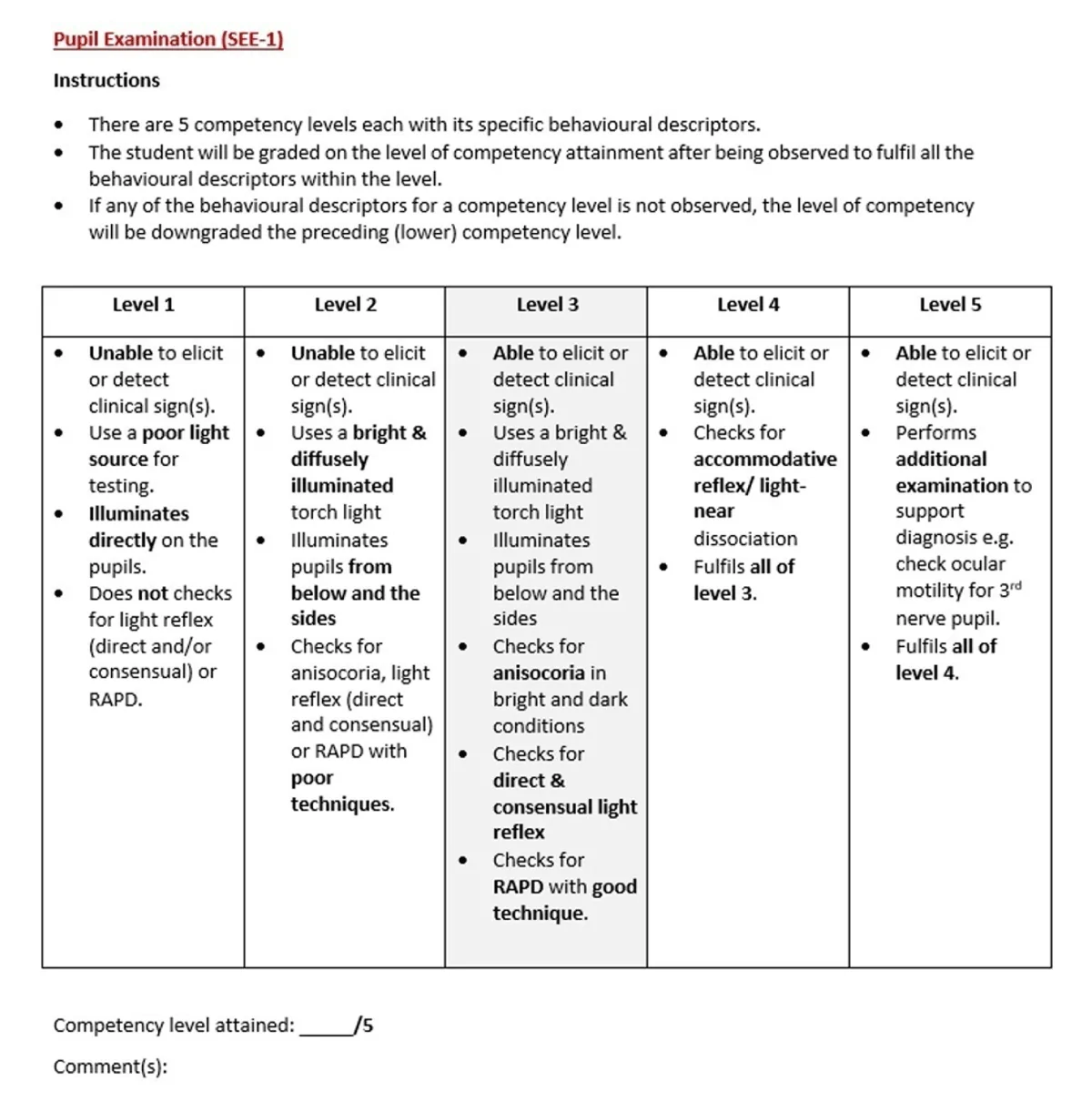
Assessment rubric for micro-clinical evaluation exercise.

**Micro-Clinical Evaluation eXercise (Micro-CEX)** (10 minutes): This workplace-based assessment evaluated the psychomotor aspect of SEE, assessing accuracy, speed, and time-sharing abilities during patient examinations. A rubric with five competency levels was used to grade performances (Figure 2).

### D. Procedures

All tests were conducted under the supervision of independent faculty members. Blinding was only partial: MCQs were auto-scored, but teachers and assessors for the End-of-Rotation Test and Micro-CEX could not be fully blinded to intervention or centre because group assignment was institution-based.

The Pre-test MCQ test was administered on the first day of the clinical rotation before either of the educational interventions was initiated in both study groups, followed by the Post-test MCQ immediately after the final session.

The End-of-Rotation Tests were graded by a senior ophthalmologist, with a second ophthalmologist reviewing for consistency. Any discrepancies in grading were resolved through discussion.

The Micro-CEX was conducted one week after the intervention, with senior ophthalmologists assessing students’ eye examination skills on real patients using a standardized, behaviourally anchored rubric. Case difficulty was not formally controlled, but the rubric assessed generic SEE performance domains rather than case-specific diagnosis, and all assessments occurred within the same clinical context. Teaching faculty were excluded from assessment to reduce expectancy effects. Remediation for underperforming students was standardized across centres and initiated only after data collection.

Faculty Contact Time was derived from the net duration of the Face-to-Face teaching session only. A research assistant timed each session with a digital stopwatch from the start of teaching to the end of the final SEE activity. The three fixed 10-minute breaks (total 30 minutes, consistent across both groups) were subtracted, so the Faculty Contact Time reflected only direct faculty–student instructional time (time taken to deliver planned content under routine conditions), excluding breaks and preparation time.

### Data analysis

Study data were keyed into a secured central database. The encrypted, anonymised data were sent to two biostatisticians for analysis using IBM®SPSS® (version 25). Statistical significance was set at p<.05. Continuous variables were tested for normality and analysed with appropriate parametric or non-parametric tests. Ordinal variables (e.g., competency levels) were analysed using non-parametric statistic.

As the SCOPE cohort was drawn from two centres whereas the F2FT cohort came from one centre, we pre-specified assessment of possible centre effects. Baseline equivalence was first checked using gender distribution and pre-test MCQ scores. Where outcomes differed between the two SCOPE centres, centre was entered as a covariate in the analysis of covariance (ANCOVA) to adjust for between-centre variation in baseline knowledge or local teaching context. Where no centre effect was detected, no adjustment was applied.

## Results

Data distribution will be presented first, followed with stratification by gender distribution and baseline knowledge between the SCOPE and F2FT groups. Hypothesis 1 will be tested using MCQ, End-of-Rotation Test, and Micro-CEX scores. Hypothesis 2 will be tested by comparing Faculty Contact Time.

### Distribution of the Collected Data

Continuous variables (MCQ scores, End-of-Rotation Test scores, Faculty Contact Time) showed no significant deviation from normality (p > .05) (Supplementary Table 1), and parametric tests were used for subsequent analysis.

### Equivalence of Both Experimental Groups

Baseline comparability was assessed by comparing gender distribution (Fisher’s exact test) and Pre-test MCQ scores (Student’s t-test) (Table 2). No significant between-group differences were detected. A baseline Micro-CEX was not performed because students had not yet been taught SEE (psychomotor domain).

**Table 2:** Baseline comparison of the gender distribution, Pre-test Multiple Choice Question Total Scores and Section-Scores (pupils, visual field, and extra-ocular movement examinations) between both experimental groups.


	SCOPE GROUP (N = 95)	F2FT GROUP (N = 51)	*P*-VALUES

**Gender**			**Fisher Exact test**

Male	44 (46.3%)	23 (45.1%)	1

Female	51 (53.7%)	28 (54.9%)	

**Pre-test MCQ Test Scores [Mean (Standard Deviation)]**			**Student’s t-test**

Total	22.31 (5.53)	22.24 (5.74)	.943

Pupillary Examination	8.16 (2.63)	7.76 (2.38)	.361

Visual Field Examination	8.26 (2.29)	8.00 (2.34)	.515

Extra-Ocular Motility Examination	5.88 (2.31)	6.47 (2.56)	.176


### Efficacy of the SCOPE Intervention to Improve Cognitive Skills

**MCQ Total Score:** The detailed breakdown of scores for both groups can be found in Table 4, and the SCOPE group had significantly higher scores in all aspects. Subgroup analysis was performed to investigate ‘centre effects’ given two different institutions involved in the SCOPE group. Within this subgroup, the student’s t-test found no significant difference in pre-test MCQ scores. However, significantly higher post-test MCQ scores were found in Centre 1 (KTPH). As such, an analysis of covariance (ANCOVA) test was performed and revealed that students from Centre 2 (NUH) had less improvement in MCQ scores compared to Centre 1 (KTPH) (p = .004), indicating a significant “centre” effect (Table 3).

**Table 3:** Pre- and post-test MCQ total scores within the SCOPE group. *Student’s t-test p-value. ^ANCOVA test statistic and p-value.


	KTPH (N = 51)	NUH (N = 44)	STATISTICAL TEST OUTCOMES	

**Pre-test MCQ Test Total Scores [Mean (Standard Deviation)]**	23.22 (5.36)	21.25 (5.6)	.085*	*F*(1, 143) = 8.47, *p = .004^*

**Post-test MCQ Test Total Scores [Mean (Standard Deviation)]**	37.31 (5.83)	32.14 (4.26)	< .001*	


**Table 4:** Comparison of post-test MCQ mean scores between F2FT and SCOPE.


	F2FT GROUP (N = 51)	SCOPE GROUP (N = 95)	

POST-TEST MCQ TEST TOTAL SCORES [MEAN (STANDARD DEVIATION)]			STUDENT’S T-TEST *p*-VALUE

Total	31.75 (5.24)	34.92 (5.75)	.001

Pupillary Examination	10.06 (2.47)	11.01 (2.26)	.025

Visual Field Examination	12.02 (1.66)	13.08 (2.31)	.002

Extra-Ocular Motility Examination	9.67 (2.73)	10.82 (2.71)	.016


**Figure 3:**
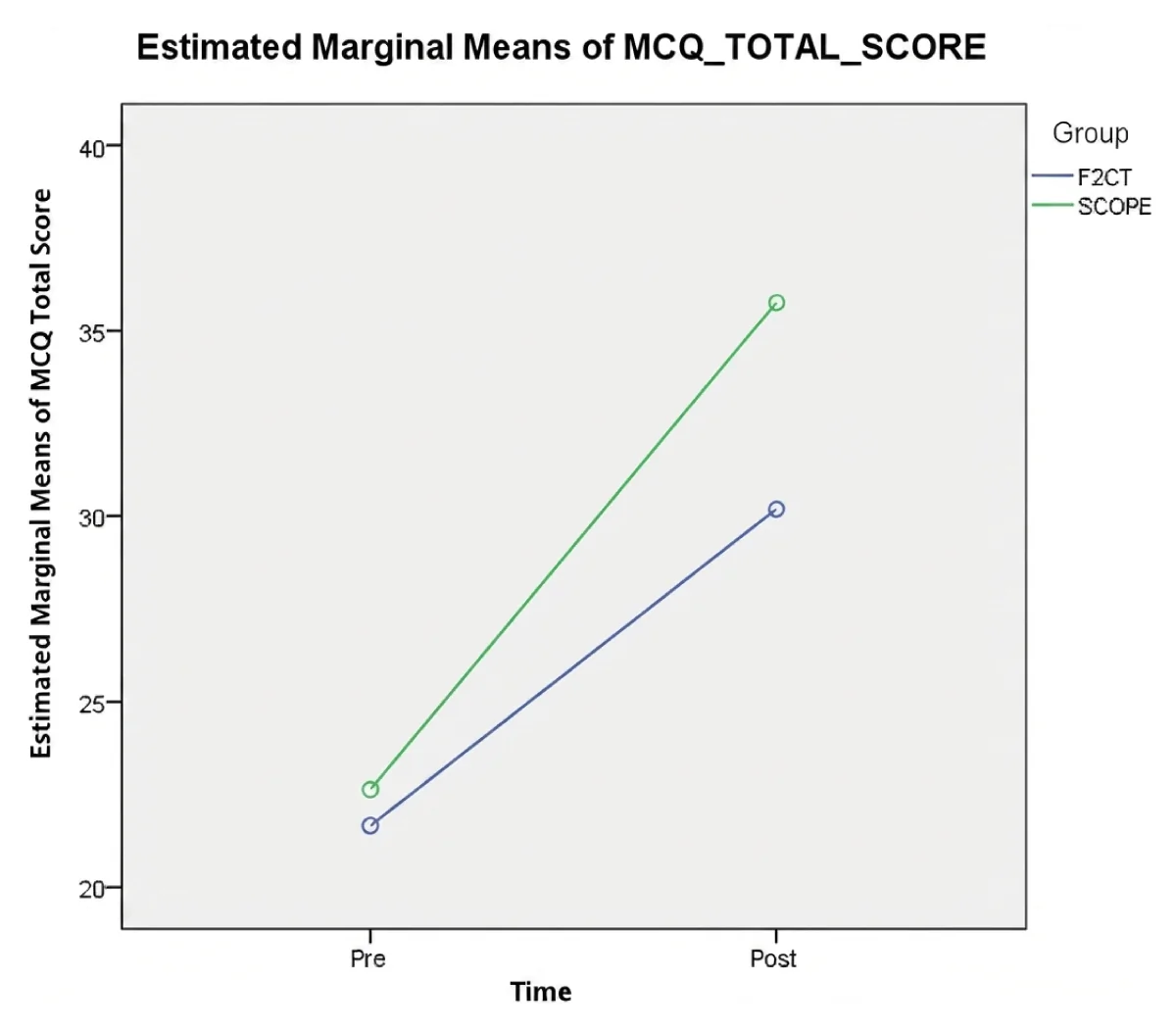
Comparison of the Estimated Marginal Means of the MCQ Test Total Score between the two experimental groups in the SCOPE group after co-variate adjustment for the ‘centre’ effect.

Repeated measures ANCOVA was thus performed to adjust for the “centre” effect when comparing between SCOPE and F2FT groups. Nevertheless, after adjustment for centre effect, the SCOPE group showed significantly greater improvement in MCQ scores than the F2FT group, with a medium effect size (F(1, 143) = 8.47, p < .001, Cohen’s d = 0.568). The greater change in the estimated marginal means with time in the SCOPE group compared to the F2FT group is graphically illustrated by a steeper slope of change in Figure 3.

**End-of-Rotation Test Score:** There was no significant difference in the mean End-of-Rotation Test Score between centre 1 and centre 2 (42.89 ± 5.68 versus 43.11 ± 5.84, *p* = .858), both of which received the SCOPE intervention. Hence, there was no need for statistical adjustment when comparing between SCOPE and F2FT groups.

The SCOPE group had significantly higher mean End-of-Rotation Test score (estimated marginal means) than the F2FT group (43.37 ± 5.85 versus 40.85 ± 6.00, *p* = .041). The effect size of SCOPE on this parameter was small (*Cohen’s d* = 0.425).

### Efficacy of the SCOPE Intervention to Improve Psychomotor Skills

The SCOPE group showed a significantly greater proportion of students achieving higher competency levels than the F2FT group for all three SEE (all p < .001) (Table 5), indicating SCOPE’s superiority in improving the psychomotor aspect of SEE.

**Table 5:** Comparing the distribution of competency levels attained on psychomotor skill assessment between the SCOPE and F2FT group.


SKILL ASSESSED	GROUP	COMPETENCY LEVEL					TOTAL	FISHER’S EXACT TEST P-VALUE

		1	2	3	4	5		

Pupillary Examination	SCOPE	0	0	5 (5.3%)	3 (3.2%)	87 (91.6%)	95	<.001

	F2FT	0	0	22 (43.1%)	3 (5.9%)	26 (51.0%)	51	

Visual Field Examination	SCOPE	0	0	9 (9.5%)	8 (8.4%)	78 (82.1%)	95	<.001

	F2FT	0	1 (2.0%)	21 (41.2%)	7 (13.7%)	22 (43.1%)	51	

Extra-Ocular Motility Examination	SCOPE	0	0	6 (6.3%)	2 (2.1%)	87 (91.6%)	95	<.001

	F2FT	1 (2.0%)	0	33 (64.7%)	9 (17.6%)	8 (15.7%)	51	


### Effect of the SCOPE Intervention on the Faculty Contact Time

The SCOPE group had significantly shorter Faculty Contact Time than the F2FT group (94.94 ± 27.30 minutes vs. 152.42 ± 23.09 minutes, p < .001), with a large effect size (Cohen’s d = 2.230), saving 57.48 ± 25.60 minutes (95% CI: 38.69, 76.26).

## Discussion

SCOPE is a novel amalgamated FC-MR model designed to teach SEE to medical undergraduates during their Ophthalmology clinical rotation. SCOPE involves student-directed e-learning using instructional videos and VMEs, followed by faculty-directed Face-to-Face learning, with guided MR before physical practice.

### Cognitive and psychomotor gains

Our results support the hypothesis that SCOPE is more efficacious than F2FT in our context, with improved cognitive (MCQ and End-of-Rotation Test scores) and psychomotor performances (Micro-CEX). These findings align with previous studies showing that e-learning and feedback enhance clinical skills [33,34]. SCOPE integrates e-learning with MR, reinforcing skill acquisition [7]. It follows Deliberate Practice Theory, offering well-defined tasks, repetitive practice through MR, and detailed feedback. Priming by FC learning materials further deepens Face-to-Face learning, making SCOPE superior to F2FT [10].

SEE are well-defined tasks, and SCOPE uses instructional videos to teach them. Instructional videos of experts performing SEE, combined with MR, offer benefits like physical training [35]. VMEs present problem-solving steps, showing the given state (e.g., small left pupil, droopy eyelid), solution (history taking, pupil exam), goal (diagnosis of lung tumour), and management process. These process-oriented examples equip students with the prerequisite knowledge for effective practice and Face-to-Face learning, unlike F2FT [36].

To achieve expertise, learners must practice intentionally, repetitively, and consciously [37]. SCOPE addresses this by providing expert-narrated MR audio files and allows students to engage in MR before viewing VMEs, optimizing anticipatory reasoning [38]. MR files can be replayed, promoting skill mastery [39]. Over time, students may execute MR independently, but MR cannot fully replace physical practice [24].

Detailed feedback prevents error perpetuation, common in novices [5]. Deliberate practice with frequent feedback improves psychomotor skills and enhances performance accuracy by reducing attentional lapses [40,41]. Faculty in SCOPE provide timely, constructive feedback to correct discrepancies and propose remedial actions. The shifting of SEE transmission to e-learning allows Face-to-Face time to focus on the intricacies of SEE performance, enabling faculty to provide detailed, corrective feedback within time constraints. In F2FT, more faculty feedback time is needed due to the lack of prior learning, but this is often limited in busy clinics.

SCOPE’s use of short, modular videos and repeatable guided mental rehearsal supports learners in managing complex skills without overwhelming them, an intentional design based on cognitive load principles [43]. Cognitive load distribution is therefore tailored to learners’ expertise [42], making it flexible for diverse competency levels. Future studies may explore cognitive load measurement using objective parameters like pupil dilation [44].

### Efficiency and faculty time

We also support the hypothesis that SCOPE requires less Faculty Contact Time than F2FT, saving nearly one hour while achieving superior learning outcomes. However, this metric captured only direct teaching time and did not include faculty preparation. F2FT used a standardized script and trained assessors, and SCOPE’s e-learning assets were developed once and reused, potentially amortizing preparation over cohorts. Thus, SCOPE likely lowers ongoing FCT while front-loading design time. Future work should quantify total faculty effort (development, updating, delivery) to compare models comprehensively. By pre-loading knowledge and enabling self-practice, SCOPE permits richer supervision/feedback in class. Students appreciate reduced teaching time and more self-directed learning, improving well-being and reducing distress [45]. SCOPE can enhance student satisfaction while easing teaching workload. SCOPE promotes self-directed learning, which is essential for professional development and high-quality learning [46,47,48]. Students take more responsibility for their learning, developing the ability to self-assess and adjust their performance [49]. This is crucial in medicine, where tutors are often busy clinicians. SCOPE supports self-directed learning by providing necessary materials and skills.

### Study Limitations

This study has several limitations. First, allocation by training centre produced a quasi-experimental, cluster-allocated design with partial blinding, introducing potential selection bias and limiting causal inference. To mitigate this, students followed pre-assigned rotations independent of investigators, baseline equivalence (gender, Pre-test MCQ) was demonstrated, standardized assessment procedures and behaviourally anchored rubrics were used, and primary analyses were adjusted for centre where appropriate. Although a centre effect appeared on one metric (MCQ Total Score), SCOPE’s advantage persisted after adjustment across outcomes. Future cluster-randomized or stepped-wedge trials with stronger blinding procedures are needed to confirm causality.

Second, total learner time was not recorded, and time-on-task was not measured. Although SCOPE e learning duration was fixed and matched F2FT objectives, unmeasured out-of-session self-learning (including additional e learning or MR) could have contributed to between group differences; thus improvements may reflect combined effects of design, time, and spacing. Nevertheless, SCOPE’s compressed design uses an intense mode of delivery [50] with clear instructional design, well sequenced assessments with timely feedback, and active student communication to optimize time-on-task. Future time-matched, exposure-controlled studies with learning analytics are needed to isolate modality and sequencing effects.

Third, to limit differential exposure, SCOPE e-learning was confined to a single 3-hour window and matched the objectives demonstrated live in F2FT; both arms used the same VMEs with in-session replay. We did not record learner time outside scheduled sessions; future time-matched, exposure-controlled trials are warranted to disentangle time, spacing, and modality effects.

Fourth, factors like motivation were not measured. Motivated students may have invested more learning effort, leading to better outcomes despite similar Time-on-Task. A qualitative study could offer insights into factors influencing SCOPE’s effectiveness.

Fifth, baseline mental imagery capabilities were not assessed. Mental imagery aids motor learning by improving the accuracy of imitated movements [50], and students with poorer imagery skills might not benefit as much. Mental imagery can improve with training [51], suggesting that the quality of MR practice may improve as students undergo multiple rounds of MR. Future studies should explore the relationship between mental imagery and performance to better understand its impact on the FC-MR model.

Lastly, the Hawthorne effect may have influenced participant behaviour [52]. While research suggests this effect is modest in healthcare settings [53], we took measures, including standardized faculty scripts, marking rubrics and students’ clarity on study procedures, to minimize bias.

### Practical Implications

SCOPE may be a useful interim strategy when F2FT is disrupted, such as during the COVID-19 pandemic, when medical students in Singapore were unable to access healthcare institutions for clinical learning. In such circumstances, the online components of SCOPE and structured MR may be preferable to no skills preparation, as they can help sustain engagement and reinforce procedural knowledge. However, these components are not a substitute for the face-to-face practice and feedback required for full skills acquisition. MR should therefore be regarded as an adjunct that primes and consolidates learning until in-person training can safely resume.

Although evaluated in a two-week rotation, SCOPE’s time-bounded, modular design (3-hour e-learning plus a single F2F session) and reduced Faculty Contact Time suggest feasibility in shorter or integrated curricula; future studies should assess dose–response and implementation fidelity across different program structures.

Although the present study focuses on ophthalmology, the FC-MR framework may be transferable to other medical subspecialties requiring integration of cognitive and psychomotor learning (e.g., neurology, otorhinolaryngology, procedural medicine). Its short, modular design and reduced faculty demands may also make it adaptable to compressed or resource-limited clinical rotations and useful for remote or hybrid learning environments, such as those necessitated by pandemic-related disruptions.

In conclusion, this study suggests that the FC-MR model can support cognitive and psychomotor learning while improving faculty time efficiency. The novel contribution of this work lies in showing that integrating FC and MR within a single, theory-informed framework may maintain comparable or better learning outcomes with less Faculty Contact Time.. By combining structured pre-class learning with guided mental practice and targeted feedback, the SCOPE model operationalises deliberate practice principles more comprehensively than either method alone.

**Supplementary Table 1:** Normality tests for the Pre-test and Post-test Multiple Choice Question Test (Total) Scores, End-of-Posting Test Scores, and Faculty Contact Time in both experimental groups.


	NORMALITY TESTS AND *P*-VALUES			
	
	SCOPE GROUP (N = 95)		F2FT GROUP (N = 51)	
	
	SHAPIRO-WILKTEST	KOLMOGOROV-SMIRNOV TEST	SHAPIRO-WILKTEST	KOLMOGOROV-SMIRNOV TEST

**Pre-test MCQ (Total) Test Score**	.663	.387	.387	.557

**Post-test MCQ (Total) Test Score**	.378	.327	.327	.797

**End-of-Posting Test Score**	.093	.432	.432	.886

**Faculty Contact Time**	.913	.402	.402	.653

